# CD25 Appears Non Essential for Human Peripheral T_reg_ Maintenance In Vivo

**DOI:** 10.1371/journal.pone.0011784

**Published:** 2010-07-30

**Authors:** Marie-Ghislaine de Goër de Herve, Emmanuel Gonzales, Houria Hendel-Chavez, Jean-Luc Décline, Olivia Mourier, Karim Abbed, Emmanuel Jacquemin, Yassine Taoufik

**Affiliations:** 1 Unité d'Immunologie Biologique, CHU Bicêtre, Le Kremlin-Bicêtre, France; 2 Faculté de Médecine, INSERM 10-12, Le Kremlin-Bicêtre, France; 3 Faculté de Médecine, Université Paris-Sud, Le Kremlin-Bicêtre, France; 4 Service d'Hépatologie Pédiatrique et Centre de Référence National de l'Atrésie des Voies Biliaires, CHU Bicêtre, Le Kremlin-Bicêtre, France; New York University, United States of America

## Abstract

**Background:**

IL-2 has been reported to be critical for peripheral T_reg_ survival in mouse models. Here, we examined T_reg_ maintenance in a series of paediatric liver transplant recipients who received basiliximab, a therapeutic anti-CD25 monoclonal antibody.

**Methodology/Principal Findings:**

FoxP3^+^ CD4 T cells were analyzed by flow cytometry before liver grafting and more than 9 months later. We found that *in vivo* CD25 blockade did not lead to T_reg_ depletion: the proportion of FoxP3^+^ cells among CD4 T cells and the level of FoxP3 expression were both unchanged. IL-2Rβ expression was enhanced in FoxP3^+^ cells both before and after basiliximab treatment, while the level of IL-2Rγ expression was similar in T_regs_ and non-T_regs_. No significant change in the weak or absent expression of IL-7Rα and IL-15Rα expression on FoxP3^+^ cells was observed. Although the proportion of FoxP3^+^ cells among CD4 T cells did not vary, food allergies occurred more rapidly after liver grafting in patients who received basiliximab, raising questions as to T_reg_ functionality in vivo in the absence of functional CD25.

**Conclusions:**

CD25 appears non essential for human T_reg_ peripheral maintenance in vivo. However, our results raise questions as to T_reg_ functionality after therapeutic CD25 targeting.

## Introduction

Basiliximab is a humanized therapeutic monoclonal antibody targeting the alpha chain of the IL-2 receptor (CD25) and used in combination to induce immunosuppression during solid organ transplantation [1]. Basiliximab binding can completely block the interactions of IL-2 with IL-2Rα [2]. The IL-2Rα amino acids responsible for IL-2 interaction overlap largely with the basiliximab epitope [2], [3].The binding affinity of basiliximab to IL-2Rα (0.14 nM) appears much higher than that of IL-2 to IL-2Rα (10 nM) [2], [3]. This therapeutic antibody targets activated CD4 T cells that transiently express CD25, thereby preventing IL-2-mediated T cell proliferation. However, regulatory CD4 T cells also express CD25 constitutively. Regulatory CD4 T cells are subdivided into natural and adaptive T_regs_ [5]. Forkhead transcription factor (*FoxP_3_*) gene expression is required for their development and function [6]. T_regs_ have suppressive effects on T and B cells in humans and mice [6]. Multi-organ autoimmune disorders occur when this population is removed from normal mice and when FoxP*_3_* is mutated in both humans and mice [6]. T_regs_ also inhibit T cell responses to tumours and pathogens [7], [8]. Finally, T_regs_ play a key role in allograft acceptance. The indirect pathway of allo-recognition is particularly important for the allo-tolerance role of T_regs_ [9]. Interleukin-2 has been reported to be involved in T_reg_ maintenance [6], [10], in keeping with the tolerogenic properties of this cytokine. Here, we examined the in vivo effect of CD25 blockade by basiliximab on T_regs_ in young children undergoing liver transplantation.

## Results and Discussion

### No significant change in the FoxP3^+^ CD4 T cells subset following in vivo CD25 blockade

**Figure 1:**
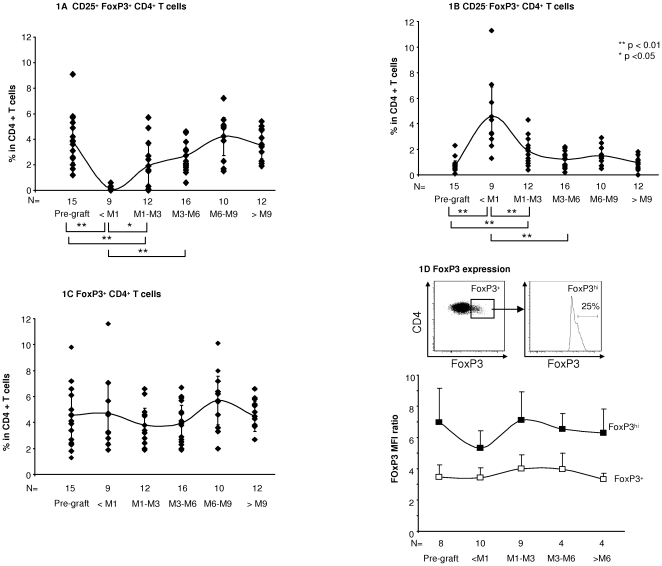
No significant effect of a blocking anti-CD25 antibody on the proportion of human FoxP3^**+**^ CD4 T cells in vivo. The proportion of CD25^+^FoxP3^+^ (**1A**), CD25^−^FoxP3^+^ (**1B**) and total FoxP3^+^ cells (**1C**) among CD4 T lymphocytes was determined before liver grafting and basiliximab injection, and for more than 9 months thereafter; data are means (curves) and SEM at the different time points. In **1D**, the FoxP3^+^/FoxP3^−^ and FoxP3^hi^/FoxP3^−^ MFI ratios were used to compare the level of intracellular FoxP3 expression (see Methods). Means and SEM are shown. The Mann Whitney test was used for statistical analysis. The number of patients tested at each time point is indicated. Only statistically significant differences are indicated.

After two injections of basiliximab, the proportion of CD25^+^FoxP3^+^ cells among CD4 T cells fell sharply, while the proportion of CD25^−^FoxP3^+^ cells increased and that of total FoxP3^+^ cell among CD4 T cells did not change significantly (Fig. 1A, 1B, 1C). Absolute numbers of CD25^+^FoxP3^+^CD4^+^ T cells also fell, from 23.8±7.3 cells/mm^3^ to 0.95±1.45 (mean ± sem, p<0.01), while CD25^−^FoxP3^+^CD4^+^ T cell numbers increased from 4.0±2.3 cells/mm^3^ to 25.5±13.4 (p<0.01). The absolute number of FoxP3^+^ CD4^+^ T cells did not change significantly (28.1±9.1 versus 30.4±14.4 cells/mm^3^). This suggests that basiliximab prevented binding of the anti-CD25 staining antibody by occupying CD25 or downregulating its expression [11], but that it did not lead to significant T_reg_ depletion. Following a nadir observed during the first month following liver grafting, the proportion of CD25^+^FoxP3^+^ cells among circulating CD4 T cells gradually increased, returning to pretransplantation values 3 to 6 months after basiliximab injection. This increase in CD25^+^FoxP3^+^CD4^+^ T cells from the nadir might have been due to CD25 re-expression, but de novo T_reg_ generation or T_reg_ redistribution from tissues to blood cannot be ruled out. As shown in Fig. 1D, the level of intracellular FoxP3 expression in total FoxP3^+^ cells (based on the mean fluorescence intensity) did not vary after basiliximab injection. No significant change in FoxP3 expression was found in the FoxP3^hi^ subset, which has been reported to be highly suppressive *in vitro* [12].

### A possible role for IL-2Rβ in T_reg_ maintenance

**Figure 2:**
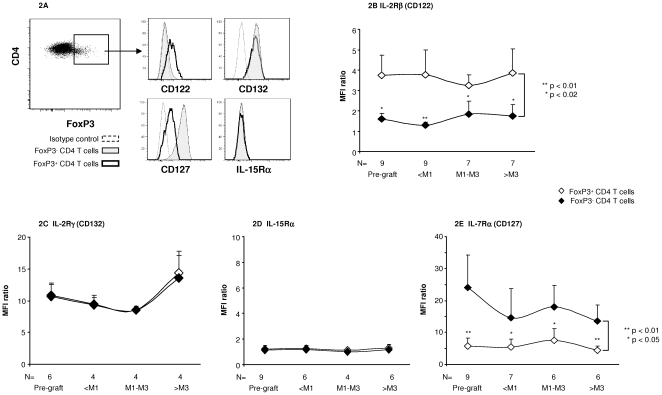
Human FoxP3^**+**^ CD4 T cells express high levels of IL-2 Rβ. IL-2Rβ (**Fig. 2A, 2B**), IL-2Rγ (**Fig. 2A, 2C**), IL-15Rα (**Fig. 2A, 2D**) and IL-7Rα (**Fig. 2A, 2E**) expression was analyzed by flow cytometry in FoxP3^+^ and FoxP3^−^ CD4 T cells before liver grafting and basiliximab injection (Figs. 2B to 2E), and at various times thereafter (2B to 2E). Fig. A shows a representative pre-graft staining profile. Figs. 2B to 2E represent the mean (SEM) MFI ratios (see Methods). The Wilcoxon test was used to compare FoxP3^+^ and FoxP3^−^ CD4 T cells. The Mann Whitney test was used to analyze the time course of expression in a given cell subset. Only significant differences are indicated.

Although the situation in blood may not necessarily reflect conditions in other tissues, basiliximab appeared to non specifically reduce the proportion of FoxP3^+^ cells among CD4 T cells. This raises several questions, including how peripheral T_regs_ can persist when deprived of functional CD25. Studies of mouse models suggest that IL-2 signalling is critical for T_reg_ homeostasis in vivo [13], [14]. The discrepancy between these reports and our present findings could be related to species differences. Alternatively, the signal mediated by the intermediate-affinity dimeric βγ IL-2 receptor may be sufficient for human T_reg_ maintenance in vivo. There are three receptor chains for IL-2, namely IL-2Rα, IL-2Rβ and γ_c_, which form different receptor complexes. A complex comprising all three subunits binds IL-2 with high affinity and is the receptor form found on activated T cells and T_regs_. IL-2Rβ and γ_c_ form an intermediate-affinity complex, in the presence of ligand, expressed on NK cells and macrophages [15], although IL-2Rβ alone has low affinity for IL-2 and γ_c_ alone has no detectable affinity [15]. Heterodimerization of IL-2Rβ and γ_c_ in the presence of IL-2 is necessary and sufficient for effective signalling, via activation of JAK1 and JAK3 kinases associated with the intracellular domains of IL-2Rβ and γ_c_, respectively, and also for STAT5 activation, which is the main pathway through which IL-2R contributes to T_reg_ maintenance [10], . We examined IL-2Rβ and γ chain expression on FoxP3^+^ cells following basiliximab administration. Both at baseline and after basiliximab administration, FoxP3^+^ CD4 T cells consistently expressed significant levels of CD122, while IL-2Rβ expression was very low in FoxP3^−^ CD4 T cells (Fig. 2A, 2B). By contrast, IL-2Rγ was clearly expressed by FoxP3^+^ and FoxP3^−^ CD4 T cells, with no significant difference in the expression level between the two subsets (Fig. 2A, 2C). Following basiliximab administration, CD122 and γ chain expression by T_regs_ and non T_regs_ remained stable (Fig. 2B, 2C). The results suggest either that IL-2 promotes T_reg_ maintenance via the dimeric βγ receptor, or that another cytokine is involved, such as IL-15, which also signals via CD122 and the γ chain, and activates STAT5 [10]. Interestingly, mice lacking CD25 display a slight decrease in Tregs within the thymus but not in the periphery, while mice deficient in CD122 show a profound reduction in both thymic and peripheral Tregs [17]. Recent findings also suggest that weak IL-2 receptor signalling is sufficient to support peripheral T_reg_ homeostasis [18]. CD122 expression by Tregs may enable the persistence of an IL-2 signal sufficient for Treg maintenance following both therapeutic CD25 blockade and IL-2 suppression by tacrolimus. Previous findings show that T cell clonal expansion requires sustained IL-2R signalling, which may imply continued expression of all three IL-2R subunits [18], [19]. This could explain how anti-CD25 prevents T cell activation in vivo without affecting T_reg_ peripheral homeostasis.

### No significant change in IL-15Rα or IL-7Rα expression on FoxP3^+^ CD4 T cells following basiliximab administration

As mentioned above, another possibility is that T_regs_ use a substitute cytokine such as IL-15 when CD25 is unavailable. We found no significant IL-15Rα expression on the patients' T_regs_ before or after basiliximab injection (Fig. 2D). However, IL-15Rα differs from IL-2Rα, in that it alone binds IL-15 with high affinity, and IL-15 has been shown to act through a phenomenon of trans-presentation, in which IL-15 bound to IL-15Rα on one cell presents IL-15 and interacts with CD122 and the γ chain on another cell [20], [21]. We also examined whether T_regs_ use another T cell survival-associated cytokine such as IL-7, that shares with IL-2 the common γ-chain [20]. Human T_regs_ express low levels of IL-7Rα, the specific receptor chain for IL-7 [22], [23] (see also Fig. 2A). Following basiliximab treatment, we observed no significant change in CD127 expression on FoxP3^+^ cells, which might reflect increased use of IL-7 by T_regs_ after basiliximab therapy (Fig. 2E). However, in mice, the low level of IL7Rα expressed by Tregs may participate in the survival of FoxP3 ^low^ CD4 T cells in situations of IL-2 signalling deprivation [24].

### Basiliximab and food allergy

Another important issue is whether T_reg_ immunosuppressive functions are maintained during therapeutic CD25 blockade. This question cannot be examined in vitro, owing to the limited size of blood samples obtained from young children. Among the mechanisms by which T_reg_ cells might suppress conventional FoxP3- T cells, T_reg_ competition for IL-2 may deprive effector CD4 T cells of IL-2 signalling, thereby inhibiting their proliferation and triggering apoptosis [25]. However, peripheral Foxp3^+^CD4^+^ T cells from *Il2^−/−^* or *Il2rα^−/−^* mice have a suppressive capacity similar to that of wild-type T_regs_, suggesting that IL-2 signalling is not required for T_reg_ function [13], [14]. Also, recent studies of adult renal transplant recipients treated with basiliximab suggest that Treg suppressive functions, tested in vitro, are maintained [26], [27]. However, these in vitro results may not accurately reflect Treg responsiveness in vivo, as previously reported for Treg proliferative capacity [28]. No autoimmune disorders have so far been observed in our series of pediatric transplant recipients, and none have been reported in other series of pediatric transplant recipients who received basiliximab during induction therapy [29], [30]. Accumulating evidence points to a role of Tregs in immune tolerance of allergens [31]. Severe food allergies have been reported in a familial form of IPEX syndrome, in which the FoxP3 gene is mutated [32]. We have observed food allergies in 28% of 100 liver transplant recipients treated with tacrolimus and basiliximab, 24% of 42 comparable patients treated with tacrolimus and steroids (no significant difference) and 4.5% of 336 comparable patients treated with cyclosporine, steroids and azathioprine (p<0.01, Mann Whitney test). Interestingly, while the frequency of food allergies was not significantly different between the patients receiving tacrolimus and basiliximab and those receiving tacrolimus and steroids, allergy emerged significantly more rapidly in patients treated with tacrolimus and basiliximab (15 months [6 days-47 months] vs 36 months [15 days-104 months], mean and range, p<0.05, Mann Whitney test). The time to allergy emergence after liver transplantation was not significantly different between patients receiving tacrolimus and steroids and those receiving cyclosporine, steroids and azathioprine (38 months [15 days-85 months]; p<0.01 *vs* tacrolimus- and basiliximab-treated patients, Mann-Whitney test). Food allergies are frequent in paediatric liver graft recipients treated with tacrolimus [33], for unclear reasons. Among other possible explanations, it has been suggested that tacrolimus may increase intestinal permeability and exposure to dietary antigens while young transplant recipients are emerging from the immune anergy associated with chronic liver failure [34]–[36]. We cannot rule out the possibility that induction therapy with basiliximab may hasten the onset of clinical allergic manifestations through possible effects on T_reg_ function and therefore on Th-2 responses to allergens.

### Conclusion

We show that therapeutic CD25 blockade does not impair the survival of human peripheral FoxP3^+^ CD4 T cells, including FoxP3^hi^ cells. This could be because IL-2 Rβ chain expression by Tregs enables IL-2 binding, thus providing, together with the γ chain, the necessary survival signal. Alternatively, FoxP3^+^ cells may use other cytokines such as IL-15 or IL-7 to activate STAT5 and provide survival signals, although we found no change in the normally weak or absent expression of the corresponding specific receptors at the Treg cell surface after basiliximab administration. Although the proportion of FoxP3^+^ cells among CD4 T cells did not vary, we found that food allergies occurred more rapidly after liver grafting in young children who received basiliximab, raising questions as to Treg functionality after therapeutic CD25 targeting.

## Methods

### Ethics statement

For the biological study, data were analyzed anonymously. No samples were conserved. Written informed consent was obtained from the children's parents. The Bicêtre hospital local ethics committee waived the need for study approval. For the study of food allergy frequencies following transplantation, data were analyzed anonymously. The Bicêtre hospital local ethics committee waived the need for informed consent (which would be impossible to obtain, the study having started in 1987) and ethics committee approval for this retrospective observational study.

The biological study involved 102 children undergoing liver transplantation (mean age 29 months; range: 6–77) at Bicêtre university hospital from January 2007 to June 2009. The blood samples used here were collected for routine haematological and immunological tests prior to transplantation and during monitoring, which included flow cytometric analysis of T cell subsets.

The indications for transplantation included biliary atresia, Alagille syndrome, fulminant hepatitis, hepatoblastoma, and glycogenosis type IV. Tacrolimus (Prograf®, Astellas), a calcineurin inhibitor, was administered orally at an initial dose of 0.1 mg/kg per day; the dose was then adapted to achieve blood trough levels of 8–12 ng/ml during the first month post-transplantation and 5–8 ng/ml thereafter. Basiliximab (Simulect®, Novartis Pharma S.A.) was administered intravenously at a unit dose of 10 mg (body weight <35 kg). All the patients were given a first dose within 8 hours following graft reperfusion and a second dose on day 4 post-transplantation.

Circulating Treg lymphocytes were analyzed before transplantation and during the first year after transplantation in 500-µl blood samples. Cells were stained in whole blood. Intracellular FoxP3 expression in CD4^+^ T lymphocytes was analyzed by flow cytometry (FacsCanto, Beckton Dickinson) after surface staining with anti-CD4-PC5 (Beckman Coulter), anti-CD3 APC-Cy7 (eBioscience) and anti-CD25 APC (Beckton Dickinson), as well as anti-CD122 (IL-2Rβ)-PE (Beckman Coulter), anti-CD127-PE (Beckman Coulter), anti-IL-15 Rα-PE (eBioscience), or anti-CD132 (IL-2Rγ)-biotin, followed by streptavidin-PE (eBioscience). For FoxP3 labelling, blood cells were lysed (Cell BD Pharm Lyse™) then permeabilized (eBioscience FoxP3 Staining Buffer Set) before intracellular staining with anti-FoxP3-FITC (clone PCH 101, eBioscience). FoxP3^+^ cells and CD25^+^ cells were determined on the basis of the isotype control in the CD3^+^CD4^+^ lymphocyte gate. The expression levels of intracellular FoxP3 and cell-surface IL-2Rβ, IL-2Rγ, IL-7Rα and IL-15Rα in FoxP3^+^ CD4 T cells were determined on the basis of the mean fluorescence intensity (MFI). MFI data were normalized between experiments by determining the MFI ratio (MFI of the positive population/MFI of the negative population with reference to the isotype control). The FoxP3^hi^ gate was defined on the basis of the last quartile (≥75%) of FoxP3 fluorescence values in the FoxP3^+^ gate.

The retrospective observational study of food allergy frequencies following liver transplantation involved 478 children (mean age: 40 months, range: 3–182 months) who were grafted at Bicêtre hospital from January 1987 to December 2006. The indications for transplantation were similar to those indicated above. Therapeutic immunosuppression was based on tacrolimus and basiliximab in 100 patients, tacrolimus and corticosteroids in 42 patients, and cyclosporine, corticosteroids and azathioprine in 336 patients. Tacrolimus and basiliximab were administered as described above. Cyclosporine, corticosteroids and azathioprine were administered as previously reported [37]. Food allergies were diagnosed on the basis of clinical findings, skin tests, and detection of specific IgE. Clinical features included one or several of the following manifestations: lip oedema (66%), angioedema (38% of patients), urticaria (17%) and anaphylactic shock (6%). Allergens included one or several of the following: peanut (38%), egg (30%), hazelnut (25%), fish (21%), cow milk (13%), lentil (11%), wheat (9%), soya (9%), pea (8%), mustard (8%), kiwi (6%), tomato (6%), shrimp (2%), cheese (2%), chicken (2%), rice (2%), and eggplant (2%).

The Mann-Whitney and Wilcoxon tests were used for data analysis.
